# A Klinefelter boy with congenital adrenal hyperplasia: too much or too little androgens?

**DOI:** 10.1186/s13052-018-0485-x

**Published:** 2018-04-03

**Authors:** Giada Zanella, Gianluca Tornese, Elisabetta Mascheroni, Elena Faleschini, Alessandro Ventura, Egidio Barbi

**Affiliations:** 1 Department of Pediatrics, Institute for Maternal and Child Health IRCCS, “Burlo Garofolo”, Via dell’ Istria 65/1, Trieste, Italy; 2 University of Trieste, Trieste, Italy

**Keywords:** Klinefelter, Congenital adrenal hyperplasia, Hypogonadism

## Abstract

**Background:**

The simultaneous occurrence of Klinefelter Syndrome (KS) and Congenital Adrenal Hyperplasia (CAH) is an exceptional event: there are just three case reports (two children and a 51 years old man) describing males affected by both KS and 21OHD (21-hydroxylase deficiency) CAH, the first causing androgen deficiency, the latter leading to androgen excess.

**Case report:**

We report the 4th case of association of KS and CAH in a young man with CAH with good androgen control and with normal secondary sex characteristics, whose Klinefelter syndrome was diagnosed because of reduced testicular volume. He was the first reported case of association of KS and CAH who started androgen replacement therapy in the pubertal age and whose pubertal development was described and followed up step by step.

**Conclusion:**

In a boy with CAH and small testicular volume, it’s important to consider that hypogonadism may be masked by the adrenal androgens excess and a karyotype should be performed once testicular adrenal rests have been ruled out.

## Background

In males with congenital adrenal hyperplasia (CAH), the fertility rate is reduced compared with the healthy population and the most frequent cause is testicular adrenal rest tumor. However high levels of adrenal androgens may hide hypogonadism.

We report a case of a boy with CAH caused by a 21-hydroxylase deficiency (21OHD) and small testicular volumes in which a chromosomal analysis revealed the association of Klinefelter syndrome (47, XXY).

## Case presentation

A CAH was suspected when he was two-years-old because of an increased growth rate with advanced bone age, precocious pubarche, and acne. The diagnosis of CAH was then confirmed by a pathologic ACTH stimulation test and by the genetic analysis, revealing a steroid 21OHD (classic form) due to a compound heterozygosity: he inherited the paternal chromosome carrying the point mutation A/CG intron 2 (655) and the maternal chromosome with the point mutation Ile-Asn-17 (999).

He started replacement treatment with oral hydrocortisone and fludrocortisone acetate with good compliance. Biochemical and somatic parameters were monitored every 3 months, adjusting the therapy to gain adequate androgen control. Some follow-up controls revealed adrenal androgens excess.

Child’s development proceeded regularly, but he didn’t start gonadal puberty. When he was 16 years-old, his testicular volume was still of 3–4 ml, assessed both using orchidometer and scrotal ultrasound. Testes ultrasound showed no signs of adrenal rest tumor.

He had wholly developed pubic hair and a pubertal penis length. He also had a family history of delayed puberty and his bone age was delayed by 3 years. Blood examination revealed low basal testosterone (1.82 ng/mL), normal basal FSH (10.1 mUI/mL) and basal LH (7 mUI/mL).

LH peak after GnRH stimulation test was 34.9 mUI/ml and FSH peak 16.2 mUI/ml.

A short hCG stimulation test was performed to assess the testes ability to produce testosterone: it showed a good gonadal response, with an increase in serum testosterone after hCG stimulation from a basal value of 2.45 ng/mL to 5.53 ng/ml.

Serum antimüllerian hormone (17.3 ng/mL) and serum inhibin B (44 ng/dL) were normal [1].

His brain magnetic resonance imaging was normal.

When he was 18, he reported a regular sexual activity without erectile dysfunction. His physical appearance was unremarkable, he reached his target height, and his weight and body mass index were normal, but testicular volume was still 4 ml. A new testes ultrasound showed small testis with no signs of adrenal rest tumor.

Gonadotropin levels were slightly increased (FSH 12.92 mUI/ml and LH 10.38 mUI/ml). Semen analysis showed no sperm, even after centrifugation of the specimen; there were just a few round spermatids.

Then Klinefelter Syndrome was suspected, and a karyotype was carried out: it confirmed a 47,XXY chromosomal arrangement on 50 metaphases, so Klinefelter Syndrome (KS) was diagnosed. Single Nucleotide Polymorphisms-array was normal.

## Discussion

CAH affected men have problems with maintaining fertility because of two major mechanisms: the first one is gonadotropin suppression from adrenal-derived androgens, which causes testicular atrophy and impairs spermatogenesis; the second one is the development of testicular adrenal rest tumors (TARTs), which leads to gradual destruction of the gonad. [2, 3].

KS affects 1 in 667 live male births and is the most common sex chromosome disorder. It is the clinical result of an additional X chromosome in males (47,XXY), although other chromosome abnormalities (such as 46,XY/47,XXY mosaicism; 48,XXXY; 49,XXXXY) account for 10–20% of cases. Virtually all men with KS have infertility and small testes with hypergonadotropic hypogonadism [4, 5].

The simultaneous occurrence of KS and CAH is extremely rare: there are just three case reports describing adult men affected by both KS and 21OHD CAH, the first causing androgen deficiency, the latter leading to androgen excess.

The first case was reported by Yamaguchi et al. [6] in 1994, and it is about a Japanese boy who was diagnosed as having CAH caused by 21OHD at birth, but he was untreated until age 10. He showed sexual precocity and marked acceleration in somatic growth till completely stopping growing at 9 years old, but only at 10 years old he started steroid treatment. After age 10, he was recognized as having bilateral small and firm testes: a chromosomal examination and a testicular biopsy revealed a 47,XXY karyotype, so the diagnosis of KS associated with CAH was made.

Parker et al. [7] (2006) reported the case of a premature boy with positive newborn screening for CAH, so he immediately started therapy with hydrocortisone, fludrocortisone, and sodium chloride supplementation. He had mild intrauterine growth retardation and a difficult neonatal course; genetic test confirmed 21OHD. When he was 8 months old his physical exam was notable for growth retardation, poor weight gain, microcephaly, and mild developmental delay; therefore many investigations were ruled out, also peripheral blood cell karyotype that revealed a mosaic 48,XXY + mar/47,XXY, indicating KS. Cytogenetic and molecular study showed the presence of a marker chromosome, uniparental isodisomy of chromosome 6, and two identical, maternally derived X chromosomes because of multiple episodes of nondisjunction.

The third case of association of KS and 21OHD CAH was reported by Balestrieri [8] in 2008: they described a 51-year-old man came to their attention because of mastodynia; he had bilateral gynecomastia with normal virilization and muscular masses, but both testes were small (4 mL) with a firm consistency. He was 174.5 cm tall, according to his genetic target height. He reported a usual pubertal onset and development, but he was infertile because of azoospermia documented at the age of 31 years. His blood tests showed hypergonadotropic hypogonadism and an altered adrenal function (elevated serum ACTH and 17-OH progesterone in the presence of normal urinary cortisol levels). No abnormalities of sexual behavior, in particular of libido, erectile function, and sexual intercourse, were ever reported in his life. KS and CAH resulting from 21OHD were suspected and then confirmed; in particular, karyotype analysis showed a 47,XXY polysomy, the biopsy of the testis revealed atrophied seminiferous tubules with Leydig cell hyperplasia, the ACTH stimulation test and CYP21 gene analysis were consistent with the diagnosis of nonclassical 21OHD. He was treated initially with an only corticosteroid replacement therapy, but a worsening of hypogonadism and the onset of sexual symptoms (progressive reduction of sexual desire and erectile function) occurred, then he also started androgen replacement therapy. He also underwent mammary plastic surgery.

The interest in describing our case report lies in the age and the clinical history of our patient: he is the first one whose the simultaneous occurrence of KS and CAH was diagnosed in the young adult age and whose pubertal development was followed step by step.

Unlike the Yamamughi case but similar to Parker case, our patient started steroid replacement therapy since he was a child (Parker’s patient from the newborn period, our patient from 2 years of age) with good adrenal axis control (unlike the Yamamughi case).

In the first two cases of association between KS and CAH [6, 7] previously reported in the literature, early diagnosis did not allow investigation of the adult phenotype and the pathophysiological correlates, while the third one [8] reported a 51 years old man characterized by very mild clinical features of the two syndromes that became evident only in adulthood and he had never been treated with hormonal replacement therapy before.

KS was expressed in our case with small testes and azoospermia, without clear hypergonadotropic hypogonadism and with an average virilized phenotype, a height in the genetic target and no psychosocial morbidity (he is now going to graduate in a scientific faculty at the university). He was similar to the Balestrieri’s patient, but he had a classic CAH, no gynecomastia, he was younger and he had always been treated with steroid replacement therapy.

Balestrieri et al. hypothesized that the high levels of adrenal androgens because of CAH were responsible for his patient’s normal pubertal onset, sexual behavior, normal virilization and muscular tropism in adulthood, thus counterbalancing the partial deficiency of androgens of testicular origin because of KS.

Their hypothesis that adrenal androgen excess masked the KS-related hypogonadism is supported by the quick and progressive deterioration of sexual health with the lowering of serum testosterone levels when corticosteroid therapy was started in their patient.

These considerations could not be applied entirely to our case because he had relatively reasonable control of the androgen excess since he was 2 years old (just some follow-up controls revealed adrenal androgen excess).

Perhaps, clinical expression of the association of KS and 21OHD CAH may be different in different ages, and there may be an unpredictable clinical response to replacement therapy in the various subjects. Some recent studies [9] [10] showed an association among the length of androgen receptor CAGn repeats, different phenotypes, and social characteristics in men with KS, but the role of this receptor is still unclear.

Our patient KS phenotype could be attenuated by adrenal androgen excess despite apparent good control at the 3 months follow up blood tests.

## Conclusion

This is the 4th case report of the simultaneous occurrence of KS and CAH.

In a boy with CAH and small testicular volume, it’s important to consider that hypogonadism may be masked by the adrenal androgens excess and a karyotype should be performed once testicular adrenal rests have been ruled out.

When considering the frequency of both 21-OH deficiency and Klinefelter syndrome, the association between these two diseases should be expected to be higher than documented (only 3 cases reported since now).

### Copyright notice

The authors affirm that the manuscript submitted is original that all statements asserted as facts are based on authors careful investigation and research for accuracy, that the manuscript does not, in whole or part, infringe any copyright, that it has not been published in total or in part and is not being submitted or considered for publication in total or in part elsewhere.

## Abbreviations

17OHP: 17-hydroxyprogesterone
21OHD: 21-hydroxylase deficiency
ACTH: Adrenocorticotropic hormone
CAH: Congenital adrenal hyperplasia
FSH: Follicle-stimulating hormone
GnRH: Gonadotropin-releasing hormone agonist
hCG: Human chorionic gonadotropin
KS: Klinefelter syndrome
LH: Luteinizing hormone
TARTs: Testicular adrenal rest tumors
